# News and Events of the Week

**Published:** 1911-03-25

**Authors:** 


### News and Events of the Week.

A public meeting is to be held in the Mansion House, the Lord Mayor presiding, on Friday, March 31, in support of a memorial to Florence Nightingale.

The joint committee (of which Lord Crewe has acted as chairman), chosen towards the end of last year, has decided on a statue in London, and on an annuity fund for the relief of trained nurses unable to provide for their old age or infirmity, and who are in a state of destitution.

The Midwives Act of 1902 came into full operation last year; and at the recent annual meeting of the Association for Promoting the Training and Supply of Midwives high tribute was paid to the new order of midwifery and to the work of the Association, which exists to meet the requirements of the Act. Princess Christian and the Archbishop of Canterbury were re-elected President of the Council and President of the Association respectively. Mr. A. L. Leon, L.C.C. (the honorary treasurer), commented on the very small proportion of four hundred applicants to the Association which had been selected as fit persons for training. Unless something was done to enable every midwife to obtain a living wage suitable women would continue to hold back from the profession and the local authorities would be forced to take the matter in hand themselves. Princess Christian briefly replied to a vote of thanks accorded to her for presiding on the motion of Mr. Cosmo Bonsor.

The seventeenth International Medical Congress will be held in London in the summer of 1913. The exact date will be fixed by the permanent international committee, which is to meet in London on April 21 and 22 next, under the presidency of Dr. F. W. Pavy. At this meeting a list of the sections will be drawn up. Propositions relative to these sections should be addressed before April 1 to Professor H. Burger, Vondelstraat 1, Amsterdam, general secretary of the committee, or to the offices of the said committee, Hugo de Gootstraat 10, La Haye, Holland.

The second medical art exhibition, "Salon des Asclépiades," will be held at the Institut Berlitz, 31 boulevard des Italiens, Paris, from March 28 to April 9 inclusive. The exhibition will contain works in painting, sculpture, pastel, engraving, and the decorative arts, executed by medical men and students. In addition, a section will be set apart for medals and objects of art directly connected with medicine. Dr. P. Rabier, 3 rue Saint-Louis-en-l'Ile, Paris, will answer all inquiries with reference to the exhibition.

Deputy-Inspector-General Matthew Coates, M.D., of Burlesdon House, Dawlish, Devon, Deputy-Inspector-General of Hospitals and Fleets, who died on January 13, left estate of the gross value of £12,734, with net personalty £9,906.

The Glamorgan County Council on Thursday last week appointed Dr. David James Morgan, of Swansea, medical officer of health and school medical officer for the county, in succession to the late Dr. Williams. The salary is £800 a year.

A competition for not fewer than twenty commissions in the Royal Army Medical Corps will be held on July 26, 1911, and following days. Applications to compete should be made to the Secretary, War Office, not later than July 17.

The Isle of Wight County Council decided last week to appoint Mr. A. M. Barford, of Chichester, medical officer, Board of Education, to the offices of county medical officer and schools medical inspector at an inclusive salary of £500. The vacancy arose through Mr. Gibson, the present schools medical inspector, declining to accept the joint appointment on the ground that the salary was inadequate. He was supported in his action by the local members of the medical profession and by a section of the Council. For the same reason two medical contemporaries declined to advertise the appointment; only twelve applications were received, and nine of the applicants withdrew before appointment.

Sir William Collins, M.D., M.S., B.Sc. Lond., F.R.C.S. Eng., has been appointed by the London County Council to the Senate of the University of London for a period of five years.

Two lectures on the "Ear" will be delivered by Dr. Dan McKenzie on Tuesday, March 28, and Friday, March 31, at 3.45 p.m., at the Central London Throat and Ear Hospital, Gray's Inn Road, W.C.

The following are the arrangements for next week at the Medical Graduates' College and Polyclinic, 22 Chenies Street, W.C.: Monday, March 27, at 4 p.m., Dr. Willmott Evans, Clinique (Skin); at 5.15 p.m., Mr. Arbuthnot Lane, "Intestinal Stasis." Tuesday, at 4 p.m., Dr. William Hunter, Clinique (Med.); at 5.15 p.m., Dr. A. F. Tredgold, "The Borderland of Insanity." Wednesday, at 4 p.m., Mr. J. Thomson Walker, Clinique (Surg.); at 5.15 p.m., Mr. Herbert Fisher, "Some Cases of Lesions of the Optic Chiasma." Thursday, at 4 p.m., Mr. P. J. Freyer, Clinique (Surg.); at 5.15 p.m., Mr. A. H. Tubby, "Treatment of Scoliosis in Childhood and Adolescence." Friday, at 4 p.m., Dr. Dan Mackenzie, Clinique (Ear, Nose, and Throat).

A lecture by Dr. Ralph Vincent on "The Production of Pure Milk: An Account of the Methods employed at the Infants' Hospital Farm," has just been published in pamphlet form. The pamphlet (price 6d.) is on sale for the benefit of the hospital, and may be obtained from the Secretary, the Infants' Hospital, Vincent Square, Westminster, London.

There were forty-one cases of measles admitted to the hospitals of the Metropolitan Asylums Board during Monday, the total being one of the highest since the outbreak. These included eight from Wandsworth, seven from Poplar, six from Woolwich, and five each from St. Marylebone and Bethnal Green. The total number of cases under treatment at midnight on Monday was 786.

The Education (Administrative Provisions) Bill, which has been introduced in the House of Commons by Mr. Tyson Wilson, seeks to impose upon medical officials the duty of saying whether a child is underfed or not. It provides that the medical inspector must examine a child alleged to be suffering from underfeeding and present an annual report on the physical condition of children attending school in his area.

